# Plasma engraved Bi_0.1_(Ba_0.5_Sr_0.5_)_0.9_Co_0.8_Fe_0.2_O_3−δ_ perovskite for highly active and durable oxygen evolution

**DOI:** 10.1038/s41598-019-40972-1

**Published:** 2019-03-12

**Authors:** Juan Sun, Zonghuai Zhang, Yansheng Gong, Huanwen Wang, Rui Wang, Ling Zhao, Beibei He

**Affiliations:** Department of Material Science and Chemistry, Engineering Research Center of Nano-Geomaterials of Ministry of Education, China University of Geosciences, Wuhan, 430074 China

## Abstract

The development of highly active and cost-effective catalysts based on noble metal free oxygen electro-catalysis is critical to energy storage and conversion devices. Herein, we highlight a plasma-treated Bi_0.1_(Ba_0.5_Sr_0.5_)_0.9_Co_0.8_Fe_0.2_O_3−δ_ perovskite (denoted as P-Bi_0.1_BSCF) as a promising catalyst for oxygen evolution reaction (OER) in alkaline media. H_2_/Ar plasma engraving could significantly increase electrochemically active O_2_^2−^/O^−^ concentration and tune the electronic structure of Co ions efficiently, and consequently tailor the intrinsic electrocatalytic ability for OER. Of note, P-Bi_0.1_BSCF, with unique crystalline core/amorphous shell structure, exhibits an enhanced intrinsic OER activity and higher stability than the noble metal IrO_2_ catalyst, which outperforms most of the reported perovskite catalysts. The present work provides new insights into exploring efficient catalysts for OER, and it suggests that, in addition to the extensively applied for surface treatment of various catalysts such as carbons and metal oxides, the plasma engraved perovskite materials also exhibits great potential as precious metal-free catalysts.

## Introduction

The pursuit of highly active and cost-effective catalysts is of prime significance for sustainable energy conversion and storage in order to develop renewable energy production. Implementing these emerging clean energy solutions, such as water splitting, direct solar and metal air batteries *et al*., highly relies on a variety of electrocatalytic reactions, such as oxygen evolution reaction (OER). Given that OER is in general impeded by its intrinsically sluggish kinetics due to a multistep four electron process, and consequently requires a considerable overpotential relative to its thermodynamic potential of 1.23 V vs. reversible hydrogen electrode (RHE). To address this, precious metal-based materials, e.g. RuO_2_ and IrO_2_, are currently employed as the efficient state-of-art OER catalysts^1^. However, high cost, low abundance as well as poor durability during long term operation badly hinder their widespread application.

Over these years, a tremendous number of alternatives based on non-precious metals materials have been intensively explored, including oxides (hydroxides)^2–5^, carbon (carbon based hybrid)^6,7^, metal oxides/carbon composite catalysts^8–10^, chalcogenides^11,12^ and phosphates^13,14^. Among these candidates, perovskite oxides have drawn much attention as competitive electrocatalysts due to their flexible states of transition metals, fascinating oxygen ion mobility and exchange kinetics, and unique tailorable properties^15–17^. For example, Suntivich *et al*.^18,19^ reported that Ba_0.5_Sr_0.5_Co_0.8_Fe_0.2_O_3_ (BSCF) perovskite provided a higher intrinsic OER activity in alkaline solution comparable to commercial IrO_2_ electrocatalyst, A site of Ba/Sr displays the fastest oxygen transport kinetics, while the Co-rich compositions on the B site offers faster oxygen exchange kinetic. The rational design of B-site transition metal ratio could tune the e_g_ orbital filling (σ*-orbital occupation), and thus benefited OER process^18^.

Although BSCF is known to have superior intrinsic activity, large particle sizes rendered from traditional bottom-up method^18^ limit their large-scale practical application due to low gravimetric mass activity. The poor stability caused by surface amorphization of BSCF particles under OER conditions is another tough issue for industrial application^20^. It is known that the surface area could be increased through a nanostructure strategy or surface modification, while the stability of perovskites could be improved by intrinsic methods resort such as chemical substitution or electronic configuration modification. Jung *et al*.^19^ introduced a nano-sized La_x_(Ba_0.5_Sr_0.5_)_1−x_Co_0.8_Fe_0.2_O_3_ with superior activity and stability, where the electronic states was managed by chemical substitution of A-site cations with lanthanum and particle growth was controlled by sintered temperature. Ln(Ba_0.5_Sr_0.5_)_0.5_Co_0.8_Fe_0.2_O_3_ (Ln = Nd, Sm, Gd)^21^ and Pr_0.5_(Ba_0.5_Sr_0.5_)_0.5_Co_0.8_Fe_0.2_O_3_^22^ have also been developed as highly active catalysts base on A-site chemical substitution strategy.

In this work, we highlight a feasibility of Bi introduced into A site of BSCF lattice (Bi_0.1_(Ba_0.5_Sr_0.5_)_0.9_Co_0.8_Fe_0.2_O_3−δ_, denoted as Bi_0.1_BSCF) to modify the electronic structure of perovskites. As known, bismuth oxide is usually used as an oxygen-ion conducting electrolyte in solid oxide fuel cells. Bi could also be introduced to A site of BSCF perovskite and tailor oxygen electrocatalysis ability^23^. Furthermore, surface modification is another effective strategy to improve the activity and durability of electrocatalysts. It is reported that the cold plasma process is an efficient approach for surface modification and functionalization, which can generate roughed surface, surface vacancies, defects and other active functional groups^24–28^. Therefore, for the first time, we propose the pollution-free and facile plasma modification on perovskite oxides to tailor the near-surface structure. The optimized 5% H_2_/Ar plasma-treated Bi_0.1_BSCF catalyst, which denoted as P-Bi_0.1_BSCF, demonstrates a higher intrinsic activity and durability towards OER, relative to the well-known BSCF perovskite as well as commercial IrO_2_ catalyst. An electrochemically active amorphous layer created by plasma engraving is achieved, and its effect is furthermore elaborated for OER.

## Methods

### Catalysts synthesis

Ba_0.5_Sr_0.5_Co_0.8_Fe_0.2_O_3_ and Bi_0.1_(Ba_0.5_Sr_0.5_)_0.9_Co_0.8_Fe_0.2_O_3−δ_ (denoted as BSCF and Bi_0.1_BSCF, respectively) perovskite oxides were synthesized by using a EDTA-citrate sol-gel process^17^. Briefly, the starting materials of Ba(NO_3_)_2_, Sr(NO_3_)_2_·4H_2_O, Bi(NO_3_)_3_·6H_2_O, Fe(NO_3_)_3_·9H_2_O, and Co(NO_3_)_2_·6H_2_O (Sinopharm Chemical Reagent Co., Ltd.) were mixed in deionized water in accordance with their stoichiometric amounts, employing EDTA and citric acid as chelating agents. With the introduction of aqueous ammonium hydroxide solution (NH_3_, 28%, Sinopharm Chemical Reagent Co., Ltd.) into above solution, the pH value was tuned at ~7. Such solution was stirred and then heated at 100 °C to yield a gel, then calcination at 250 °C overnight to form a solid precursor. The solid precursor of BSCF was sintered in air at 1000 °C, whereas Bi_0.1_BSCF was calcined in air at 850 °C for 4 h to obtain the powders. The commercial IrO_2_ (99.5%, Aladdin Industrial Corporation) catalysts was studied for comparison.

The freshly-prepared Bi_0.1_BSCF catalyst was subsequently conducted via a plasma cleaner (MING HENG, PDC-MG) with a gas flow of air, Ar or 5% H_2_/Ar for the plasma ignition (commercial 13.56 MHz RF source). Different irradiation time (0 min, 2 min, 3 min, 5 min and 8 min) with powers of ~130 W and pressure of 90 Pa was applied during plasma process. The optimized 5% H_2_/Ar plasma-treated Bi_0.1_BSCF for 3 min was used for detailed studies, which denoted as P-Bi_0.1_BSCF.

### Materials characterization

Phase structures of the as-prepared catalyst powders were determined by XRD on Bruker (D8 Focus, Cu Kα radiation). Program FullProf was employed for the diffraction refinement. SEM images were performed on a SU-8010 SEM, whereas high resolution TEM images equipped with EDS were conducted on a Tecnai G2 F20 U-TWIN TEM. XPS measurements of the catalysts were carried out on a Kratos Axis Ultra DLD instrument. The obtained XPS spectra were calibrated by referencing C 1 s to 284.6 eV, and simulated using the XPSPEAK41 software. We analyzed the specific surface areas by Brunauer Emmet Teller (BET) system with N_2_ as the adsorptive medium. Approximately 2.0 g samples were weighed and degassed at 250 °C for 4 h before nitrogen physisorption at the temperature of liquid nitrogen (77 K). The ability of perovskites to adsorb OH^−^ after exposure to water (100% humidity) at room temperature for 2 h was estimated from the Fourier transform infrared spectra (FTIR, Nicolet iS50, Thermo Scientific America). Oxygen temperature programmed desorption (O_2_-TPD) measurement was surveyed to analysis the oxygen desorption properties.

### Electrochemical evaluation

The as-prepared BSCF, Bi_0.1_BSCF, P-Bi_0.1_BSCF and commercial IrO_2_ (~5 μm, Sigma Aldrich 99.9%) catalysts were, respectively, ground in mortar to disperse well. The mixture, including 40 mg perovskite catalyst, 8 mg Ketjen black (KB, EC-600JD), 5 mL ethanol and 0.25 mL 5 wt.% Nafion solution were ultrasonicating to obtain a homogeneous ink. A 2 μL catalyst ink was then deposited onto the polished glassy carbon electrode surface with a uniform loading of ~0.2832 mg cm_disk_^−2^ (~0.2266 mg_catalyst_ cm_disk_^−2^). Electrodes containing IrO_2_ were also prepared with a similar loading of 0.2587 mg cm_disk_^−2^. The OER electrochemical characteristics were conducted in N_2_-saturated 0.1 M KOH electrolyte with a standard three electrode cell configuration (Pine Research Instrumentation). 1 M Hg/Hg_2_Cl_2_ electrode and platinum sheet were applied as the reference and the counter electrode, respectively. The potentials in this work were iR corrected, where the value of R is the ohmic electrolyte resistance via high frequency AC impedance. Cyclic voltammetry (CV) curves for OER were recorded on the rotating disk electrode from 1.3 to 2.0 V vs. RHE at 1600 rpm, with the scan rate of 10 mV s^−1^.

## Results and Discussion

**Figure 1:**
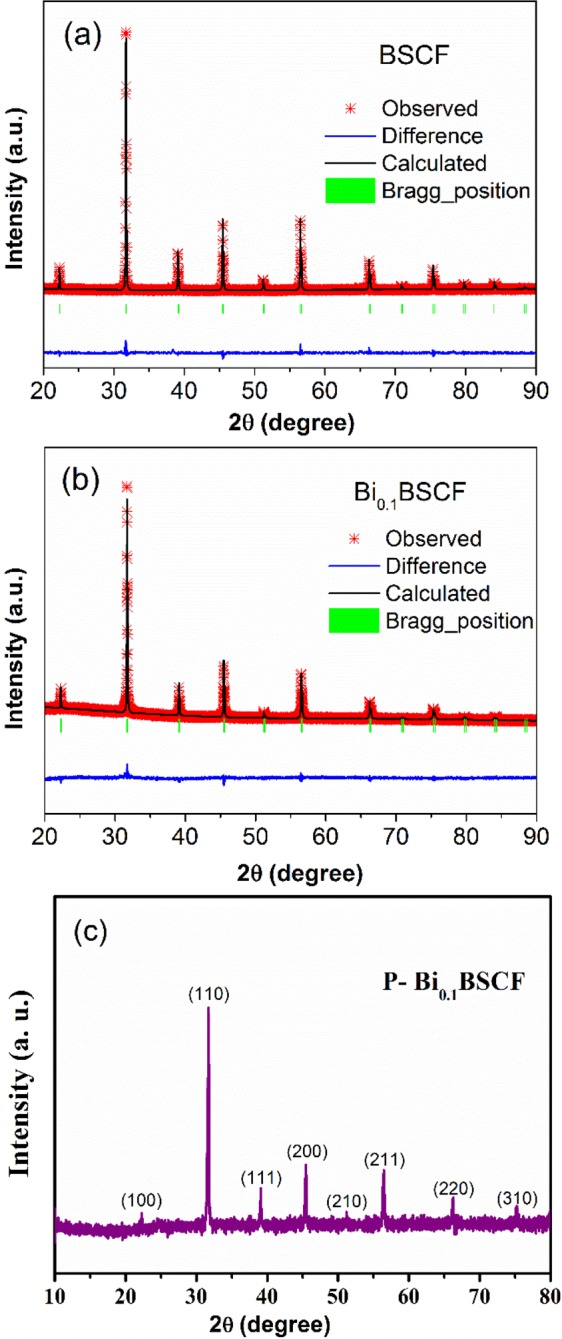
(**a**) Refined diffraction patterns of BSCF powder (**b**) Refined diffraction patterns of Bi_0.1_BSCF, (**c**) Diffraction patterns of P-Bi_0.1_BSCF.

Fig.1a,b show the Rietveld refined powder XRD pattern of pristine Ba_0.5_Sr_0.5_Co_0.8_Fe_0.2_O_3_ and Bi_0.1_(Ba_0.5_Sr_0.5_)_0.9_Co_0.8_Fe_0.2_O_3−δ_ (denoted as BSCF and Bi_0.1_BSCF, respectively). Upon Rietveld refinements, the fitted lattice parameters of BSCF and Bi_0.1_BSCF are summarized in Table S1 (ESI). One can see that both BSCF and Bi_0.1_BSCF present a cubic perovskite structure with the space group of *Pm-3m*. Schematic representation of A-site doping perovskite structure of Bi_0.1_BSCF is described in Figure S1 (ESI). The phase structure of Bi_0.1_BSCF in this study agrees well with the reported literature^23^. The typical diffraction peak (110) of Bi_0.1_BSCF moves to a higher angle compared to pristine BSCF, indicating that the introduction of Bi into BSCF lattice causes lattice contraction. This might be attributed to the substitution of the larger Sr^2+^ (0.158 nm) and Ba^2+^ (0.175 nm) ions by smaller Bi^3+^ (0.124 nm). Additionally, upon XRD analysis (Fig. 1c), no obvious structural change of P-Bi_0.1_BSCF is observed after plasma treatment for 3 min using 5% H_2_/Ar as the generating gas.

**Figure 2:**
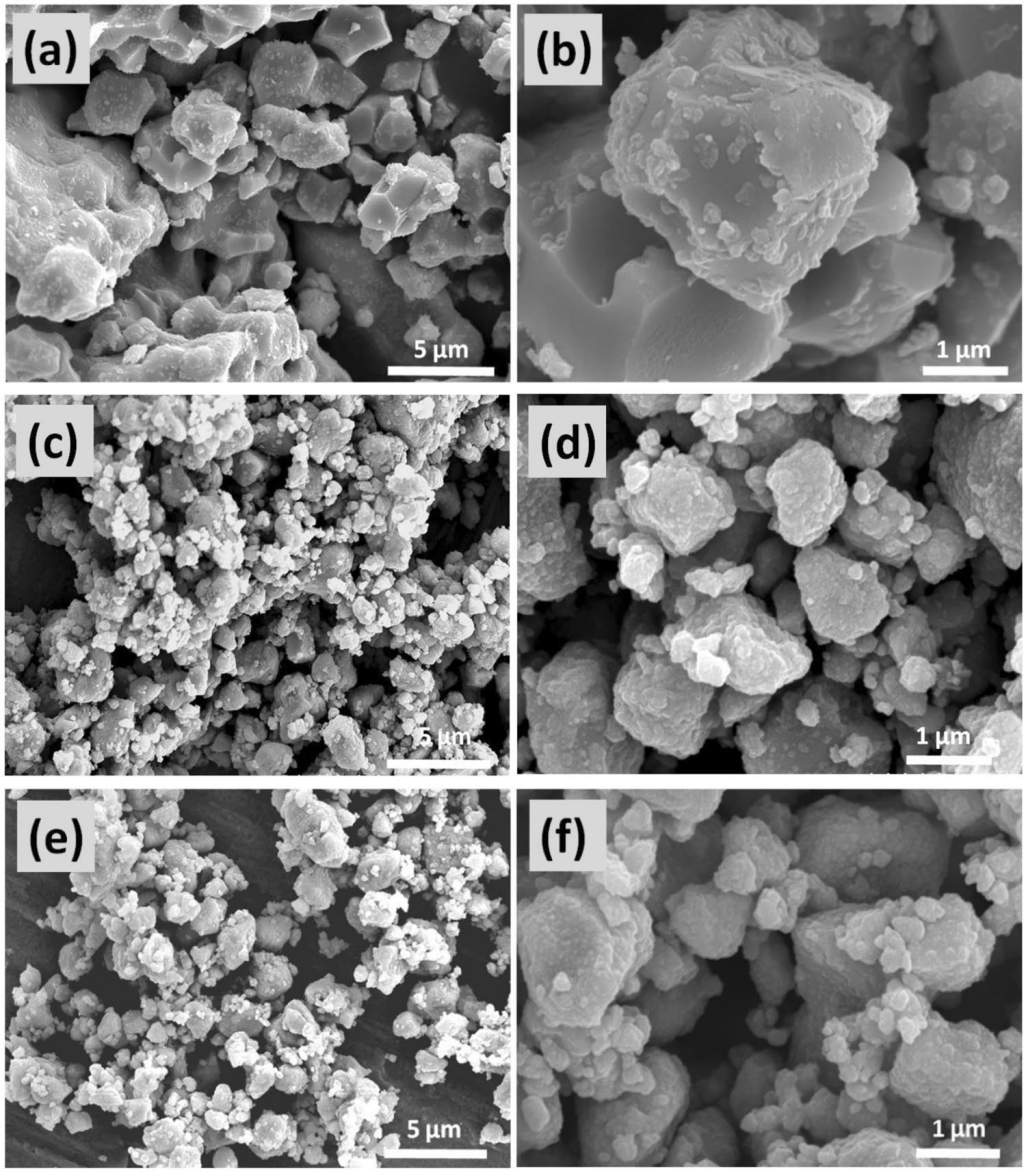
SEM images of (**a**,**b**) BSCF, (**c**,**d**) Bi_0.1_BSCF, and (**e**,**f**) P-Bi_0.1_BSCF.

Shown in Fig. 2 is the SEM morphology of the as-prepared BSCF, Bi_0.1_BSCF, and P-Bi_0.1_BSCF catalysts. It is clearly seen that the particle size of Bi_0.1_BSCF is 0.5~1 um (Fig. 2c,d) and is smaller than that of initial BSCF (Fig. 2a,b), which is probably due to its much lower sintering temperature. Accordingly, Bi_0.1_BSCF has a larger surface area of 3.0771 m^2^ g^−1^, which is 4.3 times larger than that of BSCF (0.7132 m^2^ g^−1^) as estimated via BET method (Fig. S2, ESI). Although the SEM morphology of P-Bi_0.1_BSCF does not change obviously after 5% H_2_/Ar plasma engraving (Fig. 2e,f), the BET surface area of P-Bi_0.1_BSCF increase by 14% compared to the freshly prepared Bi_0.1_BSCF. This demonstrates that the plasma treatment could engrave the surface of Bi_0.1_BSCF perovskite and thereby increase the specific surface area.

**Figure 3:**
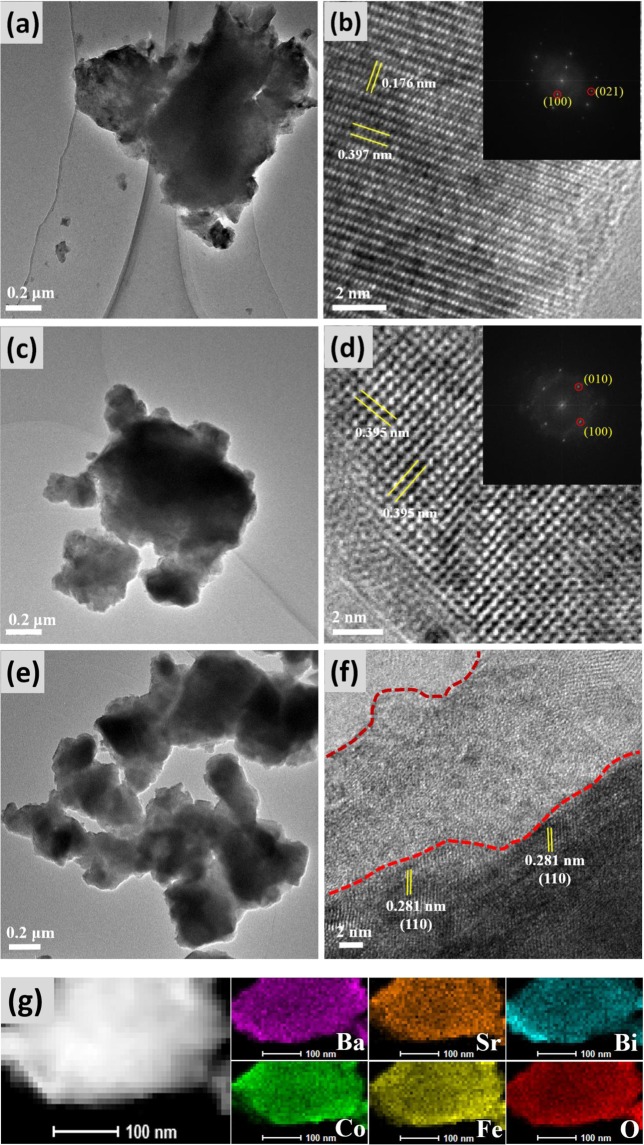
Bright-field TEM, High-resolution TEM image and corresponding fast-Fourier transformed pattern of (**a**,**b**) BSCF; (**c**,**d**) Bi_0.1_BSCF; (**e**,**f**) P-Bi_0.1_BSCF; (**g**) EDS mapping of P-Bi_0.1_BSCF.

To further investigate the crystalline structural change of BSCF after Bi-doping and plasma treatment, HR-TEM are performed on BSCF, Bi_0.1_BSCF and P-Bi_0.1_BSCF catalysts, as shown in Fig. 3. The cubic perovskite structure of BSCF and Bi_0.1_BSCF is also confirmed by Fourier transformed pattern. The lattice diffraction fringes in Fig. 3b are 0.397 and 0.176 nm, which are well indexed to the (100)_BSCF_ and (021)_BSCF_ interplanar spacing, respectively. In the case of Bi_0.1_BSCF, the lattice diffraction fringes in Fig. 3d is 0.395 nm, which agrees well with the (100) interplanar spacing. One can see that the surfaces of BSCF and Bi_0.1_BSCF particles are highly crystalline with little amorphous region. After exposure in 5% H_2_/Ar-plasma for 3 min, the perovskite structure of P-Bi_0.1_BSCF is still observed in Fig. 3f (d (110) spacing of 0.281 nm). Together with XRD characterization of plasma-engraved Bi_0.1_BSCF, the results reveal that no crystalline changes after plasma treatment. In addition, STEM and the corresponding EDS mapping analysis suggests that the elements of P-Bi_0.1_BSCF are uniformly distributed without surface segregation phenomenon after plasma engraving process. Moreover, it is apparent that P-Bi_0.1_BSCF displays a unique core-shell structure, a disordered amorphous shell with a thickness of approximately 8–10 nm is generated after plasma modification. Similar phenomenon is reported by Li and his co-workers that the TiO_2_ nanosheets shows a crystalline TiO_2_ core/amorphous TiO_2−δ_ shell structure via NH_3_-plasma surface modification with oxygen deficient on shell region^29^.

**Figure 4:**
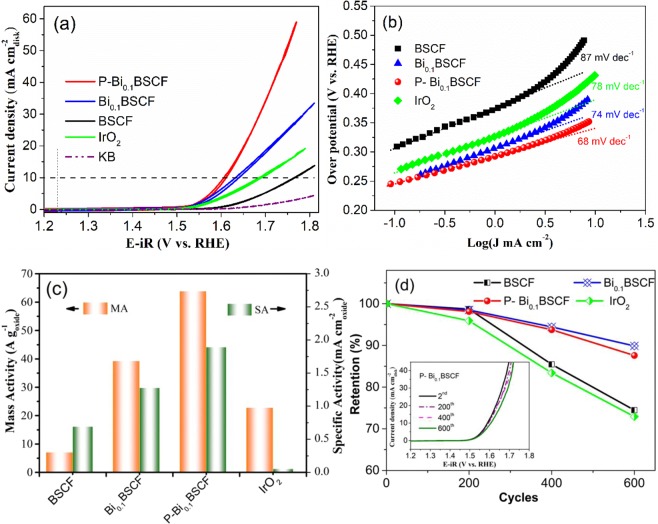
(**a**) Cyclic voltamograms of the BSCF, Bi_0.1_BSCF, P-Bi_0.1_BSCF, IrO_2_, and KB in 0.1 M KOH at 1600 rpm. (**b**) Tafel plots of these catalysts. (**c**) OER mass activity (MA) and specific activity (SA) at an overpotential of 0.4 V. (**d**) Degradation of electrochemical performance of as-prepared catalysts, inset: LSV curves of P-Bi_0.1_BSCF at 2^nd^, 200^th^, 400^th^ and 600^th^ cycles.

Figure 4a presents the CV curves of pristine BSCF, Bi_0.1_BSCF and P-Bi_0.1_BSCF catalysts under the OER potential window. Similar measurements are performed on the IrO_2_ and KB for comparison. The catalytic activity contribution of KB is subtracted according to the composition of electrode. The performance of commercial IrO_2_ catalyst is comparable to that reported elsewhere^30,31^. The CV of Bi_0.1_BSCF shows a comparably lower onset potential (1.49V) and a higher current density than the IrO_2_ and BSCF catalyst, indicating a better OER catalytic activity of Bi_0.1_BSCF. After plasma engraving, P-Bi_0.1_BSCF catalyst manifests an apparent enhancement in OER activity with a significantly improved current density. The optimized processing parameters for plasma engraving is 5% H_2_/Ar working atmosphere only for 3 min (Fig. S3, ESI). Moreover, P-Bi_0.1_BSCF catalyst exhibits a quite small overpotential (η) at a current density of 10 mA cm_disk_^−2^ of 370 mV, which is superior to that of Bi_0.1_BSCF (411 mV), BSCF (525 mV), and IrO_2_ (464 mV). Notably, the overpotential of P-Bi_0.1_BSCF favorably outperforms the reported perovskite-based OER catalysts^21,32–39^, e.g. BaCo_0.7_Fe_0.2_Sn_0.1_O_3−δ_ (410 mV)^36^, SrNb_0.1_Co_0.7_Fe_0.2_O_3−δ_ (SNCF) (500 mV)^39^ summarized in Table S2 (ESI).

Corresponding Tafel plots of the investigated samples are compared in Fig. 4b to evaluate the kinetics performance of OER. The Tafel slopes are 68, 74, 87, and 78 mV dec^−1^ for P-Bi_0.1_BSCF, Bi_0.1_BSCF, BSCF, and IrO_2_, respectively. Furthermore, the mass activity (MA) and specific activity (SA) are illustrated to evaluate the intrinsic activity, as shown in Fig. 4c. For instance, at an overpotential of 0.4 V, P-Bi_0.1_BSCF displays 8.9 times higher MA and 2.7 times higher SA relative to BSCF, respectively. The results confirm the positive contribution from the Bi-dopant and plasma engraving to the intrinsic activity. In addition,

The durability of electrocatalysts is another vital parameter for OER. In current study, we performed continuous CV curves for P-Bi_0.1_BSCF, Bi_0.1_BSCF, BSCF, and commercial IrO_2_ catalysts for 600 cycles, as shown in Fig. 4d. BSCF and IrO_2_ show a 25% and 27% reduction of its 2^nd^ activity over 600 cycles, respectively, and the poor stability of BSCF results from the leaching of A site cations to the alkaline medium^20^. In contrast to BSCF, only 10% and 12% reduction is observed in the Bi_0.1_BSCF and P-Bi_0.1_BSCF catalysts under the same condition, respectively. The TEM image of post-OER P-Bi_0.1_BSCF in Fig. S4 shows that the structure of P-Bi_0.1_BSCF is maintained and the thickness of amorphous shell did not change much after continuous CV tests compared to as-synthesized P-Bi_0.1_BSCF. In addition, the XPS spectra before and after long-term OER testing shows that the oxidation state of Bi^3+^ does not change^40,41^ (Fig. S5), which also reveals the durability of P-Bi_0.1_BSCF catalyst.

**Figure 5:**
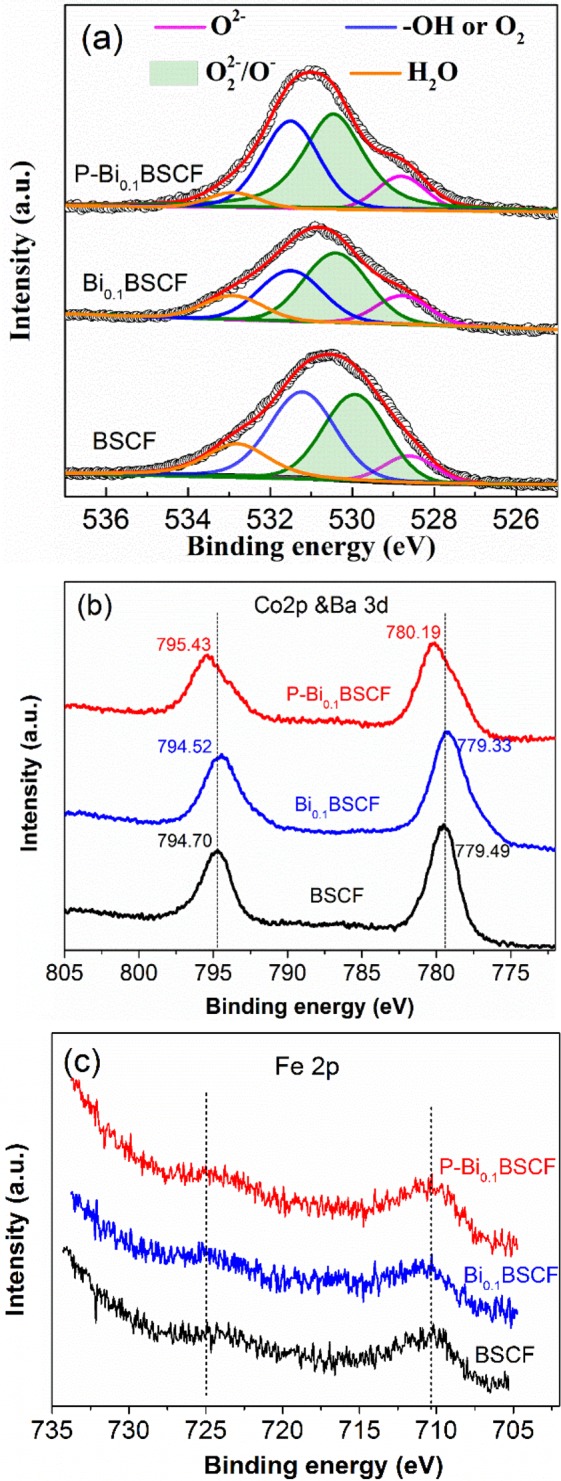
XPS spectra of (**a**) O1s, (**b**) Co 2p & Ba3d, (**c**) Fe 2p for BSCF, Bi_0.1_BSCF and P-Bi_0.1_BSCF samples.

To understand the source responsible for the excellent OER activity of P-Bi_0.1_BSCF, the surface state of BSCF, Bi_0.1_BSCF and P-Bi_0.1_BSCF catalysts is intensively studied by XPS analysis. The O1s XPS spectra for BSCF, Bi_0.1_BSCF and P-Bi_0.1_BSCF is deconvoluted to four characteristic peaks (Fig. 5a). The first one at a lower binding energy (529.6 eV) represents the lattice oxygen (O^2−^), followed by the surface oxidative oxygen O_2_^2−^/O^−^ (530.5 eV), hydroxyl groups OH^−^ or adsorbed O_2_ (531.5 eV), and adsorbed molecular H_2_O of the oxide surface (532.9 eV)^42^. The relative content of the lattice and surface oxygen species derived is listed in Table S3 (ESI). The content of surface oxidative oxygen O_2_^2−^/O^−^ increases from 35.68% with BCSF to 42.17% with Bi_0.1_BSCF. Moreover, the O_2_^2−^/O^−^ concentration is further increased to ~49.23% by plasma engraving, indicating the existence of abundant active oxygen species on the amorphous region of P-Bi_0.1_BSCF oxide surface. Importantly, the electrochemically active O_2_^2−^/O^−^ are proved to be beneficial for catalyzing OER^43,44^. Also, the highly oxidative oxygen species on the surface are reported to be closely relevant to the oxygen vacancies^45^, and surface oxygen vacancies play a key role for perovskite oxides in catalyzing OER^46^. It reveals that the plasma engraving could create more electrochemical active sites on the amorphous shell. The similar phenomenon of surface amorphization and simultaneous increased activity for efficiently electro-catalyzing OER is also found in other catalysts^47–50^.

The core-level spectra of Co 2p & Ba 3d for these three catalysts are presented in Fig. 5b. The overlapping between Co 2p and Ba 3d spectra makes it different to identify the surface Co valence by peak fitting, while the Co oxidation state could be inferred from the shift of peak position. Compared to BSCF, the positions of Co peaks for the Bi_0.1_BSCF shift to lower binding energies, suggesting that the surface valence of Co slightly decrease owing to high valence Bi-substitution. After 5% H_2_/Ar plasma engraved, the two main peaks of P-Bi_0.1_BSCF shift to higher binding energies obviously, which indicates the presence of surface cobalt in a higher oxidation state. It is reported that the high valence of Co could also play a positive role in the improvement of OER activity^51^. No distinctive variation was observed in the Fe state of catalyst surface (Fig. 5c). On the basis of the XPS results, the increased O_2_^2−^/O^−^ concentration together with electronic structure tuning of Co induced by plasma engraving might be responsible to the enhancement in OER performance.

**Figure 6:**
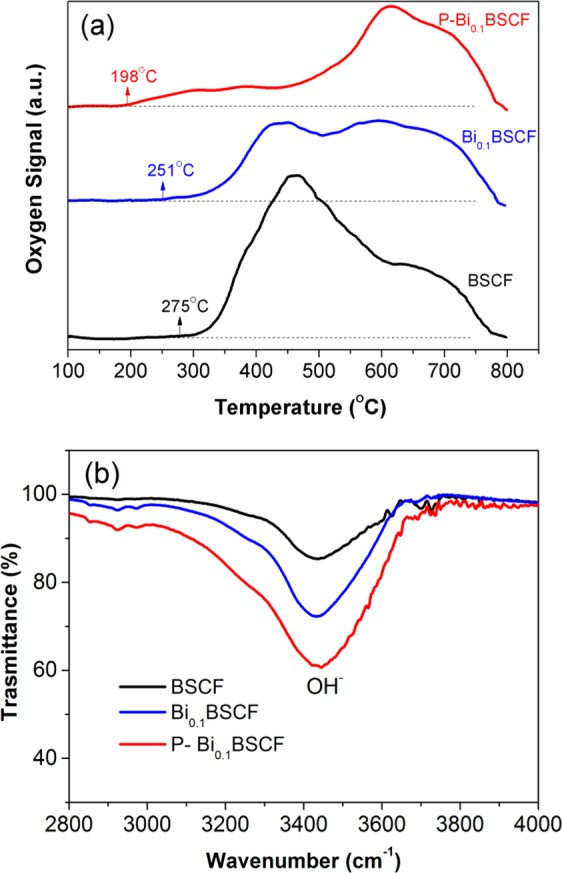
(**a**) O_2_-TPD profiles of BSCF, Bi_0.1_BSCF and P-Bi_0.1_BSCF samples. (**b**) FTIR spectra of BSCF, Bi_0.1_BSCF and P-Bi_0.1_BSCF samples after exposure to 100% humidification.

The redox property of BSCF, Bi_0.1_BSCF, and P-Bi_0.1_BSCF is studied by O_2_-TPD, as shown in Fig. 6a. The desorption peak of the Bi_0.1_BSCF catalyst, associated with the reduction of Co iron, occur at approximately 251 °C, which is lower than that of BSCF (275 °C). It’s worth noting that, after 5% H_2_/Ar plasma engraved, the desorption temperature of P-Bi_0.1_BSCF catalyst reduce remarkably to nearly 198 °C, this indicates an excellent oxygen desorption capability^35^ and reflects a higher oxygen surface exchange ability for P-Bi_0.1_BSCF perovskite. The result is consistent with the EIS analysis that P-Bi_0.1_BSCF has better ionic and charge transfer abilities. Besides O_2_ desorption capability, the P-Bi_0.1_BSCF catalyst also shows good OH^−^ adsorption. As observed by FTIR spectroscopy (Fig. 6b), a broad IR band centers at approximately 3466 cm^−1^ appeared, which corresponds to H-bonded OH^−^ stretching vibration^52^. Obviously, Bi_0.1_BSCF exhibited a stronger OH^−^ absorption ability than initial BSCF, and the OH^−^ absorption ability is further improved via plasma treatment. According to the adsorbate evolution mechanism^53^, OH^−^ absorption on the active sites of perovskites is the prerequisite for OER, and thereby the large OH^−^ absorption can continually offer raw materials for the following OER process^36^. Due to the complex interplay between oxygen deficiency and surface-active redox centre of perovskite oxides, detailed mechanistic insights into the OER electrocatalysis of P-Bi_0.1_BSCF materials in alkaline media are currently limited. Nevertheless, with the superior activity, stability and cost-effectiveness, Bi_0.1_BSCF promises a novel precious-metal-free catalyst for the alkaline OER, and plasma engraving proved to be a facile and effective surface modification method to further improve the activity.

## Conclusion

In summary, a plasma engraving strategy is, for the first time, proposed for the surface modification of perovskite materials towards electrocatalyzing OER. After 5% H_2_/Ar plasma engraving only for 3 min, an amorphous layer with abundant electrochemical active oxygen species is generated on Bi_0.1_BSCF perovskite surface. Moreover, the increased oxygen surface exchange ability and OH- absorption ability of P-Bi_0.1_BSCF are achieved. Owing to the unique properties via Bi-doping as well as plasma engraving, P-Bi_0.1_BSCF exhibits a fast kinetics process with a small Tafel slope of 68 mV dec^−1^ and high stability for OER, that is superior to most of the state-of-the-art perovskite-based electrocatalysts. The foregoing results open a new avenue for engineering perovskite oxides via plasma method for highly efficient OER, which is promising for a variety of electrochemical energy storage applications.

## Supplementary information

Supplementary Information

## References

[1] Jiao Y, Zheng Y, Jaroniec M, Qiao SZ (2015). Design of electrocatalysts for oxygen- and hydrogen-involving energy conversion reactions. Chemical Society Reviews.

[2] Wang Y (2014). Reduced Mesoporous Co3O4 Nanowires as Efficient Water Oxidation Electrocatalysts and Supercapacitor Electrodes. *Advanced Energy*. Materials.

[3] Han L, Dong S, Wang E (2016). Transition-Metal (Co, Ni, and Fe)-Based Electrocatalysts for the Water Oxidation Reaction. Advanced Materials.

[4] Zhang Z (2018). Durable oxygen evolution reaction of one dimensional spinel CoFe2O4 nanofibers fabricated by electrospinning. Rsc Advances.

[5] Zhang Z (2018). Boosting Overall Water Splitting via FeOOH Nanoflake-Decorated PrBa0.5Sr0.5Co2O5+ delta Nanorods. Acs Applied Materials & Interfaces.

[6] Zhao Y, Nakamura R, Kamiya K, Nakanishi S, Hashimoto K (2013). Nitrogen-doped carbon nanomaterials as non-metal electrocatalysts for water oxidation. Nature Communications.

[7] Mao S, Wen Z, Huang T, Hou Y, Chen J (2014). High-performance bi-functional electrocatalysts of 3D crumpled graphene-cobalt oxide nanohybrids for oxygen reduction and evolution reactions. Energy & Environmental Science.

[8] Su Y (2014). Cobalt nanoparticles embedded in N-doped carbon as an efficient bifunctional electrocatalyst for oxygen reduction and evolution reactions. Nanoscale.

[9] Wang H (2017). Cobalt ion-coordinated self-assembly synthesis of nitrogen-doped ordered mesoporous carbon nanosheets for efficiently catalyzing oxygen reduction. Nanoscale.

[10] Han X (2017). Ultrasensitive Iron-Triggered Nanosized Fe-CoOOH Integrated with Graphene for Highly Efficient Oxygen Evolution. *Advanced Energy*. Materials.

[11] Qiao X, Jin J, Fan H, Li Y, Liao S (2017). *In situ* growth of cobalt sulfide hollow nanospheres embedded in nitrogen and sulfur co-doped graphene nanoholes as a highly active electrocatalyst for oxygen reduction and evolution. Journal of Materials Chemistry A.

[12] Zhang Y (2017). Nanostructured Metal Chalcogenides for Energy Storage and Electrocatalysis. Advanced Functional Materials.

[13] Zhang X (2018). Ni(OH)(2)-Fe2P hybrid nanoarray for alkaline hydrogen evolution reaction with superior activity. Chemical Communications.

[14] Fu S (2018). Ultrafine and highly disordered Ni2Fe1 nanofoams enabled highly efficient oxygen evolution reaction in alkaline electrolyte. Nano Energy.

[15] Zhu L, Ran R, Tade M, Wang W, Shao Z (2016). Perovskite materials in energy storage and conversion. Asia-Pacific Journal of Chemical Engineering.

[16] Chen D, Chen C, Baiyee ZM, Shao Z, Ciucci F (2015). Nonstoichiometric Oxides as Low-Cost and Highly-Efficient Oxygen Reduction/Evolution Catalysts for Low-Temperature Electrochemical Devices. Chemical Reviews.

[17] Gong C (2018). Atomic layered deposition iron oxide on perovskite LaNiO3 as an efficient and robust bi-functional catalyst for lithium oxygen batteries. Electrochimica Acta.

[18] Suntivich J, May KJ, Gasteiger HA, Goodenough JB, Shao-Horn Y (2011). A Perovskite Oxide Optimized for Oxygen Evolution Catalysis from Molecular Orbital Principles. Science.

[19] Jung J-I (2016). Optimizing nanoparticle perovskite for bifunctional oxygen electrocatalysis. Energy & Environmental Science.

[20] May KJ (2012). Influence of Oxygen Evolution during Water Oxidation on the Surface of Perovskite Oxide Catalysts. Journal of Physical Chemistry Letters.

[21] Kim N-I (2017). Highly active and durable nitrogen doped-reduced graphene oxide/double perovskite bifunctional hybrid catalysts. Journal of Materials Chemistry A.

[22] Xu X (2016). A Perovskite Electrocatalyst for Efficient Hydrogen Evolution Reaction. Advanced Materials.

[23] Liu Y, Ran R, Tade MO, Shao Z (2014). Structure, sinterability, chemical stability and conductivity of proton-conducting BaZr0.6M0.2Y0.2O3-delta electrolyte membranes: The effect of the M dopant. J. Membr. Sci..

[24] Zhong W (2017). Air plasma etching towards rich active sites in Fe/N-porous carbon for the oxygen reduction reaction with superior catalytic performance. Journal of Materials Chemistry A.

[25] Liu Z (2017). *In Situ* Exfoliated, Edge-Rich, Oxygen-Functionalized Graphene from Carbon Fibers for Oxygen Electrocatalysis. Advanced Materials.

[26] Wang Y (2017). Layered Double Hydroxide Nanosheets with Multiple Vacancies Obtained by Dry Exfoliation as Highly Efficient Oxygen Evolution Electrocatalysts. Angewandte Chemie-International Edition.

[27] Xu L (2016). Plasma-Engraved Co3O4 Nanosheets with Oxygen Vacancies and High Surface Area for the Oxygen Evolution Reaction. Angewandte Chemie-International Edition.

[28] Bharti B, Kumar S, Lee H-N, Kumar R (2016). Formation of oxygen vacancies and Ti3+ state in TiO2 thin film and enhanced optical properties by air plasma treatment. Scientific Reports.

[29] Li B (2014). Highly Efficient Low-Temperature Plasma-Assisted Modification of TiO2 Nanosheets with Exposed {001} Facets for Enhanced Visible-Light Photocatalytic Activity. Chemistry-a European Journal.

[30] Wang, M. *et al*. Oxidizing Vacancies in Nitrogen-Doped Carbon Enhance Air-Cathode Activity. *Advanced materials (Deerfield Beach, Fla.)*, e1803339-e1803339 (2018). 10.1002/adma.201803339 30515889

[31] Bu Y (2017). A Highly Efficient and Robust Cation Ordered Perovskite Oxide as a Bifunctional Catalyst for Rechargeable Zinc-Air Batteries. Acs Nano.

[32] Zhang D (2015). Active LaNi1-xFexO3 bifunctional catalysts for air cathodes in alkaline media. Journal of Materials Chemistry A.

[33] Park HW (2014). Electrospun porous nanorod perovskite oxide/nitrogen-doped graphene composite as a bi-functional catalyst for metal air batteries. Nano Energy.

[34] Yu J (2016). Activity and Stability of Ruddlesden-Popper-Type Lan + 1NinO3n + 1 (n = 1, 2, 3, and infinity) Electrocatalysts for Oxygen Reduction and Evolution Reactions in Alkaline Media. Chemistry-a European Journal.

[35] Sun H (2017). B-Site Cation Ordered Double Perovskites as Efficient and Stable Electrocatalysts for Oxygen Evolution Reaction. Chemistry-a European Journal.

[36] Xu X (2016). Co-doping Strategy for Developing Perovskite Oxides as Highly Efficient Electrocatalysts for Oxygen Evolution Reaction. *Advanced*. Science.

[37] Zhou S (2016). Engineering electrocatalytic activity in nanosized perovskite cobaltite through surface spin-state transition. Nature Communications.

[38] Zhu Y (2016). Enhancing Electrocatalytic Activity of Perovskite Oxides by Tuning Cation Deficiency for Oxygen Reduction and Evolution Reactions. Chemistry of Materials.

[39] Zhu Y (2015). SrNb0.1Co0.7Fe0.2O3-delta Perovskite as a Next-Generation Electrocatalyst for Oxygen Evolution in Alkaline Solution. Angewandte Chemie-International Edition.

[40] Lee CW (2018). Selective Electrochemical Production of Formate from Carbon Dioxide with Bismuth-Based Catalysts in an Aqueous Electrolyte. *Acs*. Catalysis.

[41] Zhang Z (2016). Rational Design of Bi Nanoparticles for Efficient Electrochemical CO2 Reduction: The Elucidation of Size and Surface Condition Effects. Acs Catalysis.

[42] Zhu Y (2015). A High-Performance Electrocatalyst for Oxygen Evolution Reaction: LiCo0.8Fe0.2O2. Advanced Materials.

[43] Liu R, Liang F, Zhou W, Yang Y, Zhu Z (2015). Calcium-doped lanthanum nickelate layered perovskite and nickel oxide nano-hybrid for highly efficient water oxidation. Nano Energy.

[44] Jung J-I, Jeong HY, Lee J-S, Kim MG, Cho J (2014). A Bifunctional Perovskite Catalyst for Oxygen Reduction and Evolution. Angewandte Chemie-International Edition.

[45] Zhu J (2014). Perovskite Oxides: Preparation, Characterizations, and Applications in Heterogeneous Catalysis. Acs Catalysis.

[46] Li Z (2018). Engineering phosphorus-doped LaFeO3-δ perovskite oxide as robust bifunctional oxygen electrocatalysts in alkaline solutions. Nano Energy.

[47] Gerken JB (2011). Electrochemical Water Oxidation with Cobalt-Based Electrocatalysts from pH 0−14: The Thermodynamic Basis for Catalyst Structure, Stability, and Activity. Journal of the American Chemical Society.

[48] Risch M (2011). Nickel-oxido structure of a water-oxidizing catalyst film. Chemical Communications.

[49] Zaharieva I (2011). Synthetic manganese-calcium oxides mimic the water-oxidizing complex of photosynthesis functionally and structurally. Energy & Environmental Science.

[50] Liang H (2017). Amorphous NiFe-OH/NiFeP Electrocatalyst Fabricated at Low Temperature for Water Oxidation Applications. Acs Energy Letters.

[51] Mefford JT (2016). Water electrolysis on La1-xSrxCoO3-delta perovskite electrocatalysts. Nature Communications.

[52] Zou X (2013). Efficient oxygen evolution reaction catalyzed by low-density Ni-doped Co3O4 nanomaterials derived from metal-embedded graphitic C3N4. Chemical Communications.

[53] Rong X, Parolin J, Kolpak AM (2016). A Fundamental Relationship between Reaction Mechanism and Stability in Metal Oxide Catalysts for Oxygen Evolution. *Acs*. Catalysis.

