# Supplementary material for: Plasma engraved Bi0.1(Ba0.5Sr0.5)0.9Co0.8Fe0.2O3−δ perovskite for highly active and durable oxygen evolution
Source: Sci Rep. 2019 Mar 12;9:4210. doi: 10.1038/s41598-019-40972-1 (PMC6414661; doi:10.1038/s41598-019-40972-1)
Supplement: Supplementary file 1 — Supplementary Information [file 41598_2019_40972_MOESM1_ESM.pdf]

Electronic Supplementary Information

Plasma engraved $Bi_{0.1}(Ba_{0.5}Sr_{0.5})_{0.9}Co_{0.8}Fe_{0.2}O_{3-\delta}$ perovskite for highly active and durable oxygen evolution

Juan Sun, Zonghuai Zhang, Yansheng Gong, Huanwen Wang, Rui Wang, Ling Zhao, Beibei He*

Faculty of Materials Science and Chemistry,

Engineering Research Center of Nano-Geomaterials of Ministry of Education,

China University of Geosciences, Wuhan 430074, China.

Corresponding author. E-mail:babyfly@mail.ustc.edu.cn (B.B. He)

Table S1. Refinement parameters derived from XRD patterns.

Sample	Space group	a (Å)	b (Å)	c (Å)	V(Å^3)	R_p (%)	R_{wp} (%)
**BSCF**	Pm-3m	3.98661	3.98661	3.98661	63.359	2.95	3.95
**$Bi_{0.1}$BSCF**	Pm-3m	3.98649	3.98649	3.98649	63.354	3.65	5.55

Table S2. Comparison of the electrocatalytic activity of $Bi_{0.1}$BSCF, P-$Bi_{0.1}$BSCF and other reported excellent OER catalysts in literatures.

Catalysts	Onset potential (V vs. RHE)	E_{OER} (V) @10 mA cm^{-2}	Tafel slope (mV dec^{-1})
**P-$Bi_{0.1}$BSCF (this work)**	1.49	1.60	68
**$Bi_{0.1}$BSCF (this work)**	1.50	1.64	74
**$Ba_{0.5}Sr_{0.5}Co_{0.8}Fe_{0.2}O_{3-\delta}$[31]**	1.55	~1.75	106-
**IrO_2[30]**	1.47	1.69	100
**$LaNiO_3$[29]**	1.57	~1.85	122
**$LaNi_{0.8}Fe_{0.2}O_3$[27]**	1.61	~1.70	-
**$La_{0.95}FeO_{3-\delta}$[33]**	0.58	~1.64	82
**$La_{0.6}Sr_{0.4}CoO_3$[32]**	1.60	1.85	102
**$La_{0.5}Sr_{0.5}CoO_3$(80 nm)[32]**	1.55	~1.72	69
**$BaCo_{0.7}Fe_{0.2}Sn_{0.1}O_{3-\delta}$[31]**	1.53	~1.64	63
**$SrNb_{0.1}Co_{0.7}Fe_{0.2}O_{3-\delta}$[34]**	1.53	1.73	76
**$NdBa_{0.5}Sr_{0.5}Co_{1.5}Fe_{0.5}O_{5+\delta}$[18]**	1.50	1.62	88
**$La_{0.58}Sr_{0.4}Co_{0.2}Fe_{0.8}O_{3-\delta}$@ nitrogen-doped graphene[28]**	1.60	1.72	72
**$Ba_2Bi_{0.1}Sc_{0.2}Co_{1.7}O_{6+\delta}$[30]**	1.58	~1.72	102

Note: All the catalysts were measured in a 0.1 M KOH solution.

Table S3 XPS peak deconvolution results of O 1s core levels.

**Electrocatalyst**	**lattice O^{2-}**	**O_2^{2-}/O^-**	**$-OH/O_2$**	**H_2O**
**BSCF**	9.16 %	35.68 %	39.29 %	15.87 %
**$Bi_{0.1}BSCF$**	13.96 %	42.17 %	30.34 %	13.53 %
**$P\text{-}Bi_{0.1}BSCF$**	10.99 %	49.23 %	34.07 %	5.71 %

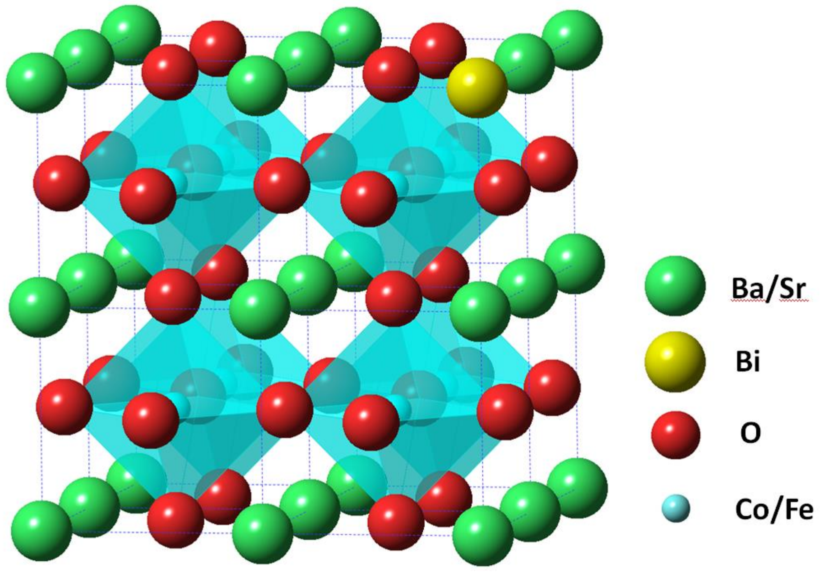

Figure S1 Schematic representation of the structure of $Bi_{0.1}(Ba_{0.5}Sr_{0.5})_{0.9}Co_{0.8}Fe_{0.2}O_{3-\delta}$ perovskite.

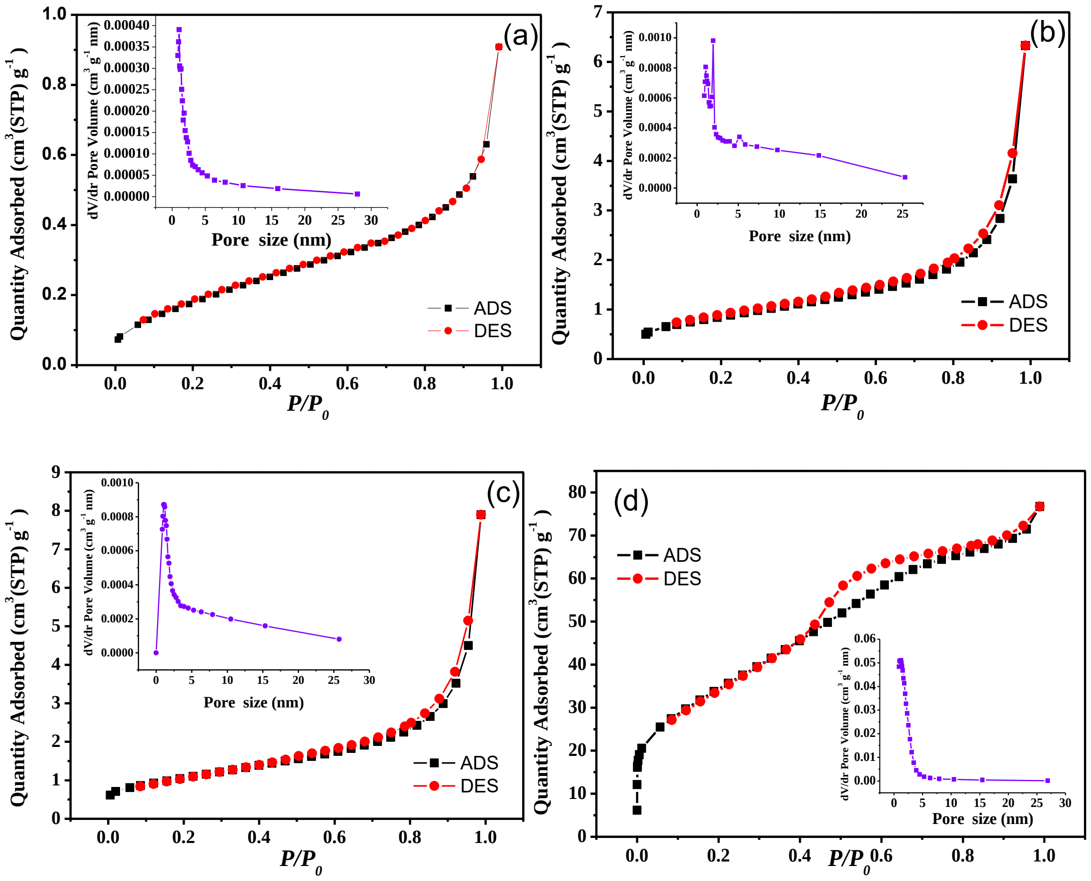

Figure S2. Nitrogen adsorption-desorption isotherms of (a) BSCF (0.7132 m^2 g^{-1}), (b) $Bi_{0.1}$BSCF (3.0771 m^2 g^{-1}), (c) P-$Bi_{0.1}$BSCF (3.5660 m^2 g^{-1}), (d) IrO_2 (125.4105 m^2 g^{-1}); the insets show the corresponding BJH pore size distribution curves obtained from the desorption branch of the nitrogen adsorption/desorption isotherms.

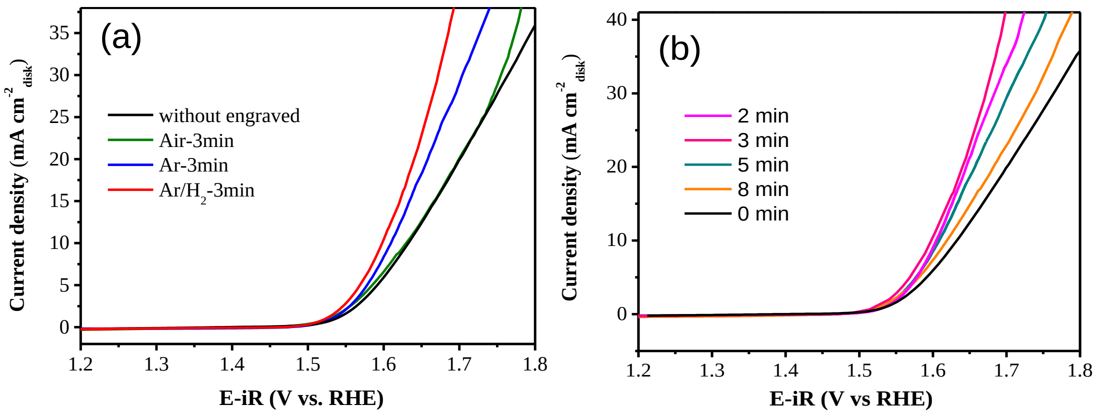

Figure S3 (a)The polarization curves of air, Ar, and 5% H_2/Ar plasma engraved for 3 min; (b) 5% H_2/Ar plasma engraved for 0 min, 2 min, 3 min, 5 min and 8 min in 0.1M KOH at a scan rate of 10 mV s^{-1}.

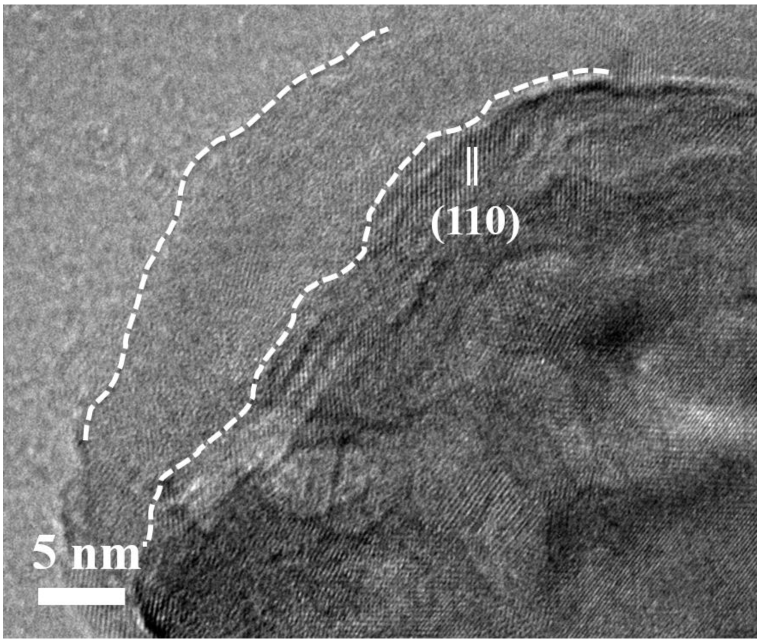

Figure S4 Near-surface TEM image of P-$Bi_{0.1}$BSCF after 600 cycles CV test.

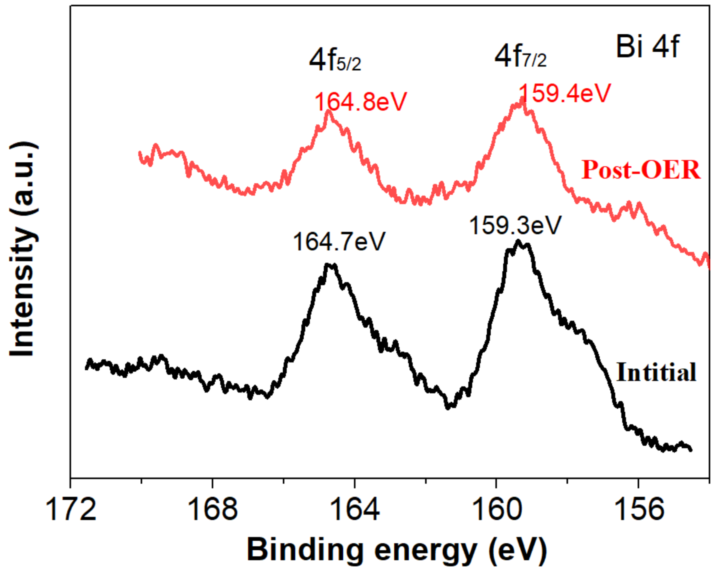

Figure S5 X-ray photoelectron spectroscopy spectrum of the Bi 4f core levels for P-$Bi_{0.1}$BSCF before and after OER test.
